# Melatonin attenuates degenerative disc degression by downregulating DLX5 via the TGF/Smad2/3 pathway in nucleus pulposus cells

**DOI:** 10.1002/jsp2.70014

**Published:** 2024-11-13

**Authors:** Kuibo Zhang, Hua Wang, Ling Mo, Xiaohui Huang, Chao Yuan, Caijun Liu

**Affiliations:** ^1^ Department of Spine Surgery The Fifth Affiliated Hospital of Sun Yat‐sen University Zhuhai China; ^2^ Department of Spine Surgery The First Affiliated Hospital of Sun Yat‐sen University Guangzhou China; ^3^ The Third Affiliated Hospital of Guangzhou University of Chinese Medicine Guangzhou China; ^4^ Guangdong Research Institute for Orthopedics & Traumatology of Chinese Medicine Guangzhou China; ^5^ Laboratory of General Surgery, The First Affiliated Hospital Sun Yat‐sen University Guangzhou China

**Keywords:** apoptosis, DLX5, intervertebral disc degeneration, melatonin, nucleus pulposus cell

## Abstract

**Background:**

Intervertebral disc degeneration (IVDD) is the leading cause of low back pain, and apoptosis plays a key role in its pathogenesis. Distal‐less homeobox 5 (Dlx5) has been reported to induce cell apoptosis. Melatonin, as a powerful antiapoptotic agent, has been widely reported.

**Aim:**

This study aimed to investigate the role of DLX5 in the pathogenesis of IVDD and the potential therapeutic role of melatonin in targeting DLX5 in IVDD.

**Materials & Methods:**

Western blotting, RT–qPCR, immunohistochemistry, si‐DLX5, Ex‐DLX5, flow cytometry, and immunofluorescence were used to examine the regulatory effect of DLX5 on apoptosis. Therapeutic efficacy was assessed by the intraperitoneal injection of melatonin into IVDD mice.

**Results:**

The expression level of DLX5 is significantly increased in IVDD, and the expression levels were positively correlated with the grade of IVDD. DLX5 was significantly upregulated in TNF‐α‐induced degenerative NP cells. Degenerative NP cells transfected with si‐DLX5 exhibited significantly less apoptosis than control cells. Melatonin significantly alleviated IVDD in surgically induced IVDD model mice.

**Discussion:**

The results revealed that the expression of DLX5 was positively correlated with the severity of IVDD and that melatonin ameliorated DLX5‐induced apoptosis and extracellular matrix imbalance by inhibiting the TGF‐β/Smad signaling pathway. This study may provide therapeutic strategies to alleviate inflammation‐induced apoptosis IVDD‐associated inflammation‐induced apoptosis.

**Conclusion:**

DLX5 plays an important role in IVDD progression by promoting apoptosis, and melatonin represents a promising therapeutic strategy for alleviating IVDD‐associated inflammation and apoptosis.

## 1 INTRODUCTION

Low back pain (LBP) is a worldwide disease that affects 11%–84% of people worldwide throughout their lifetime.^1^, ^2^ Reports estimate that LBP is one of the most common causes of disability, causing a substantial global economic and health burden.^3^ Intervertebral disc degeneration (IVDD) is the main cause of LBP and is associated with aging. Nucleus pulposus (NP) dysfunction is thought to be an IVDD‐initiating factor.^4^, ^5^ Current treatments for LBP, such as analgesics and anti‐inflammatory drugs, focus primarily on pain management and cannot delay the progression of the disease.^5^ New treatment strategies targeting IVDD are urgently needed.

The intervertebral disc (IVD) consists of an inner gelatinous nucleus pulposus (NP) and an outer fibrocartilaginous annulus (AF) that borders the superior and inferior endplates and connects the adjacent vertebral bodies. An imbalance of anabolic and catabolic processes triggers a cascade of intervertebral disc degeneration characterized by pathological changes, including increased proinflammatory cytokines, loss of the extracellular matrix (ECM), decreased numbers of nucleus pulposus (NP) cells, a shift in the cellular phenotype, cellular senescence and cell death.^6^, ^7^, ^8^, ^9^ Studies have shown that the inflammatory response of intervertebral disc cells is a key event in the progression of IVDD.^10^ A previous study revealed that inflammation can alter the microenvironment of NP cells, induce apoptosis, and ultimately lead to IVDD.^11^

The DLX family of transcription factors is known to participate in the development of multiple tissues and organs, mainly affecting the growth and differentiation of appendages, the nervous system, branchial arches, and hematopoiesis tissues.^12^ Specifically, distal‐less homeobox 5 (Dlx5), a nuclear transcription factor, plays critical roles in embryogenesis, organ development, tissue differentiation, and bone formation.^13^, ^14^ DLX5 has also been reported to be involved in cancer, organ fibril formation, and inflammation.^15^, ^16^, ^17^ Studies have shown that DLX5 is significantly upregulated in human rheumatoid arthritis and inhibits inflammation.^18^

One study showed that DLX5 was downregulated in NP cells treated with BMP‐2.^19^ However, there have been no studies on the correlation between DLX5 expression and IVDD. To investigate the roles of DLX5 in IVDD, a bioinformatic analysis was performed via public microarray datasets of IVDD obtained from the Gene Expression Omnibus. DLX5 was identified as a positive‐related mRNA in IVDD, and GO analysis revealed that DLX5 is associated with apoptosis, ECM organization, and TGF/Smad‐associated pathways. Therefore, we hypothesized that DLX5 aggravates IVDD by regulating TGF/Smad‐associated pathways to induce apoptosis.

N‐acetyl‐5‐methoxytryptamine (MEL), which is synthesized by the pineal gland and many other organs, is a neuroendocrine hormone involved in a wide range of physiological functions, including cancer‐preventing, anti‐inflammatory, antidegenerative, antioxidant, circadian rhythm regulation, and antiapoptotic activities.^20^, ^21^ Chen et al. revealed that MEL could perturb the positive inflammatory loop to alleviate IVDD.^6^ According to recent studies, the MEL‐induced TGF/Smad2/3 pathway might be a therapeutic target for oral MEL.^22^ Our pathway analysis revealed a close relationship with TGF‐β/Smad2/3. Consequently, we hypothesized that MEL affects the TGF/Smad2/3 pathway by targeting DLX5.

In summary, this study aimed to investigate the molecular mechanisms of DLX5 during the degenerative progression of IVDs. We also aimed to investigate in vitro and in vivo whether exogenous melatonin administration prevents IVDD by regulating DLX5.

## 2 RESULTS

### 2.1 Identification of DLX5 as an IVDD‐associated gene

**FIGURE 1:**
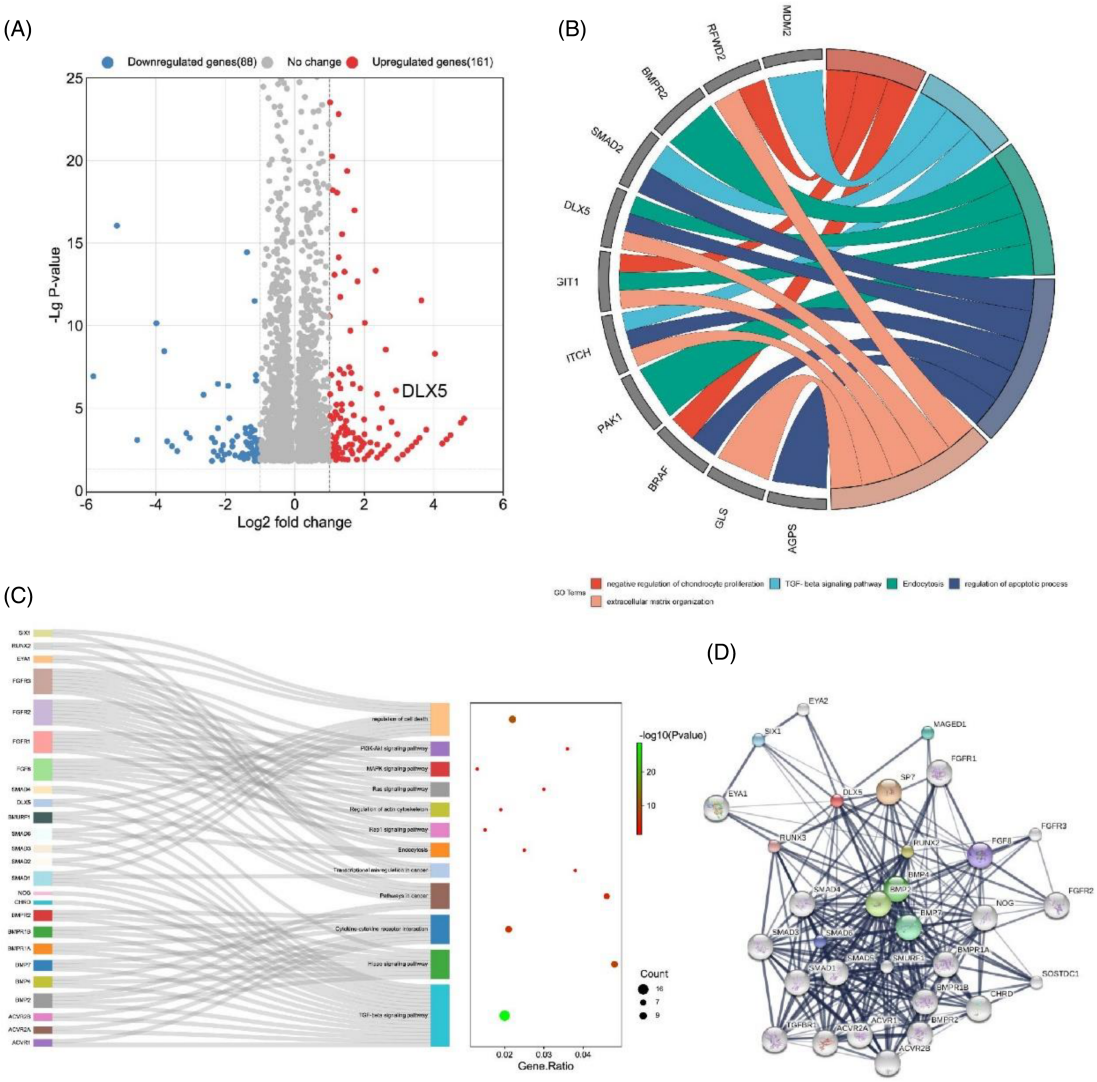
Identify of DLX5 as an IVDD‐associated gene. (A) Volcano plot depicting all abnormally expressed genes in degenerative and nondegenerative NP tissues. (B) Circos plot showing differentially expressed genes. The left column represents differentially expressed genes, and the right column represents different biological processes. (C) Sankey plot showing pathways associated with differentially expressed genes. (D) Protein interaction plot of DLX5 via the STITCH database.

First, to investigate the biological roles of differentially expressed genes in IVDD, we performed bioinformatics analysis on the Affymetrix® whole‐genome gene chip microarray GSE70362 of NP tissue samples.^23^ We identified 249 DEGs via a volcano map (upregulated: downregulated = 161:88) (Figure 1A). The GO enrichment analysis of the 249 genes revealed that DLX5 is associated with biological processes such as the TGF/beta signaling pathway, regulation of the apoptosis process, and extracellular matrix organization (Figure 1B). As a result, we speculated that DLX5 and the pathogenesis of IVDD might be closely associated. Furthermore, we conducted a Kyoto Encyclopedia of Genes and Genomes (KEGG) analysis on DLX5. We discovered that DLX5 might be involved in controlling cell death and the TGF/beta signaling pathway, similar to our earlier GO analysis (Figure 1C). Surprisingly, DLX5 was shown to interact with several proteins associated with the SMAD, BMP, and FGF protein families via gene interaction analysis on the STITCH website (Figure 1D). Overall, we hypothesized that DLX5 might be involved in the progression of IVDD by regulating TGF/β/SMAD‐associated pathways and apoptosis.

### 2.2 Upregulated expression of DLX5 in human and mouse degenerative IVDs

**FIGURE 2:**
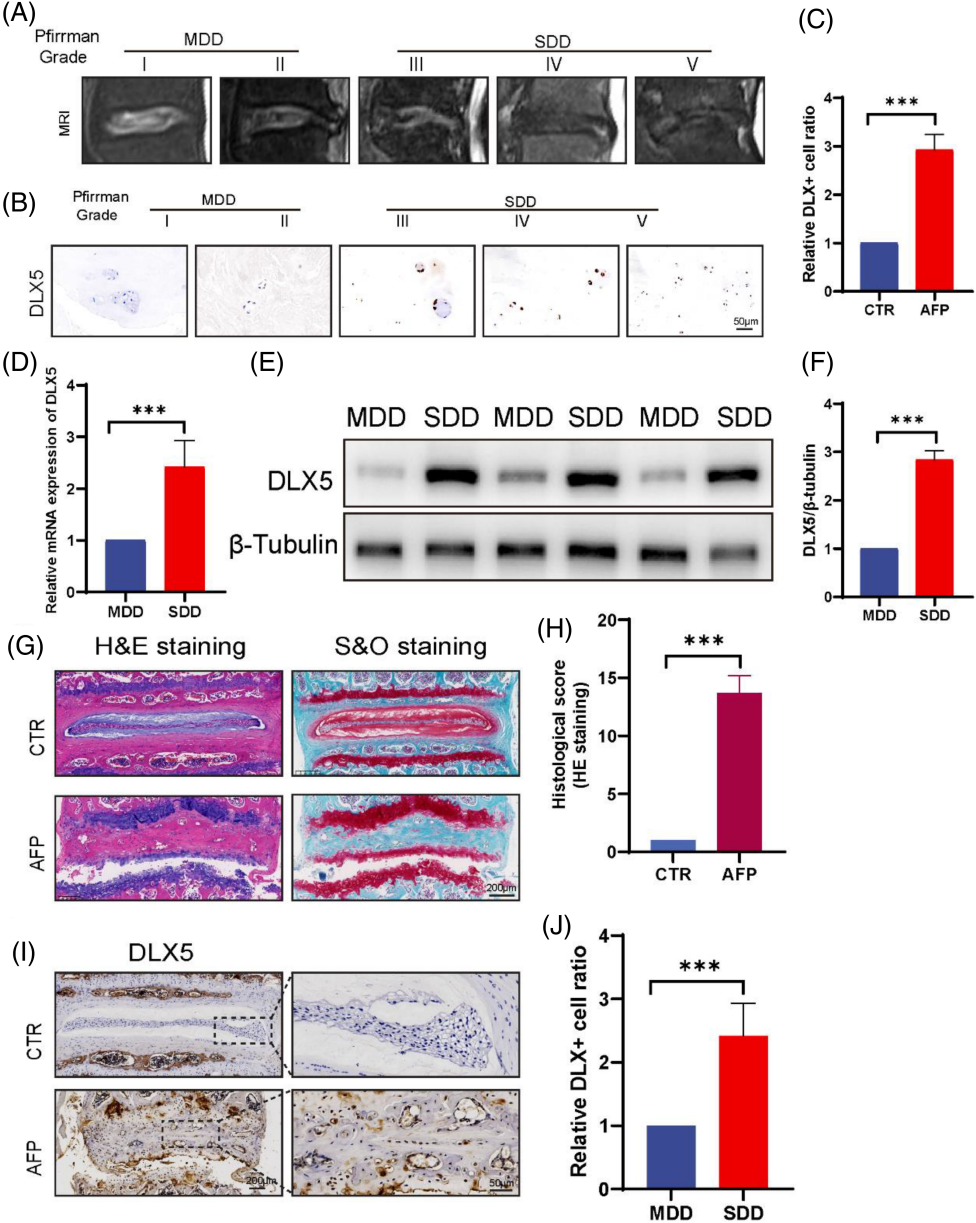
Upregulated expression of DLX5 in human and mice degenerative IVDs. Upregulated expression of DLX5 in human and mouse degenerative IVDs. (A) Representative image of five‐grade T2‐weighted MRI of patient IVDs. (B, C) IHC staining and corresponding quantitative analysis of DLX5 in each grade of NP tissue (scale bar = 50 μm). (D) RT–qPCR results of DLX5 expression in human NP tissues from MDD and SDD IVDD patients. MDD: mild degenerative disc; SDD: severe degenerative disc. (E) Western blot results of human NP tissue in the MDD and SDD groups. (F) Quantitative analysis of the Western blot results normalized to β‐tubulin. (G, H) Representative H&E and S&O images of mouse IVDs in the control and AF puncture groups. Histological scoring of H&E‐stained tissue was performed. (I, J) IHC staining of DLX5 in mouse IVDs and NP tissues and corresponding quantitative analysis. CTR: control group; AFP: annulus fibrosus puncture group. The data are expressed as the means ± SDs (*n* = 3); ***p < 0.001.

To investigate the role of DLX5 in IVDD, we collected NP tissues from patients with different degrees of human degeneration (male:female = 13:12) (Table S1, Supporting Information). Representative T2 MR images are shown in Figure 2A (MDD: mild degenerative disc; SDD: severe degenerative disc). As expected, immunohistochemical fluorescence staining and quantitative analysis revealed that DLX5 expression was significantly increased in the SDD of human tissue and that its expression level was positively correlated with the severity degree of intervertebral disc degeneration (Figure 2B,C). Similarly, RT–qPCR and Western blot results confirmed low DLX5 expression in MDD human tissues but significantly high DLX5 expression in SDD tissues (Figure 2D,E).

We also constructed a mouse IVDD model and examined the expression level of DLX5 in mouse IVDs. In the IVDD model, the structures of the NPs and AFs were mixed, and the mucopolysaccharide content of the NPs was significantly reduced (Figure 2G,H). IHC and quantitative analysis revealed that DLX5 was expressed at low levels in the normal nucleus pulposus but at significantly high levels in the AFP group in the mouse model (Figure 2I,J). In conclusion, DLX5 was significantly upregulated in both human degenerated NP tissues and mouse IVDD model NP tissues, a preliminary validation of previous bioinformatics results. These findings constitute the basis for subsequent investigations of the role and mechanism of DLX5 in IVDD.

### 2.3 The expression of DLX5 was increased in degenerative NP cells in vitro

**FIGURE 3:**
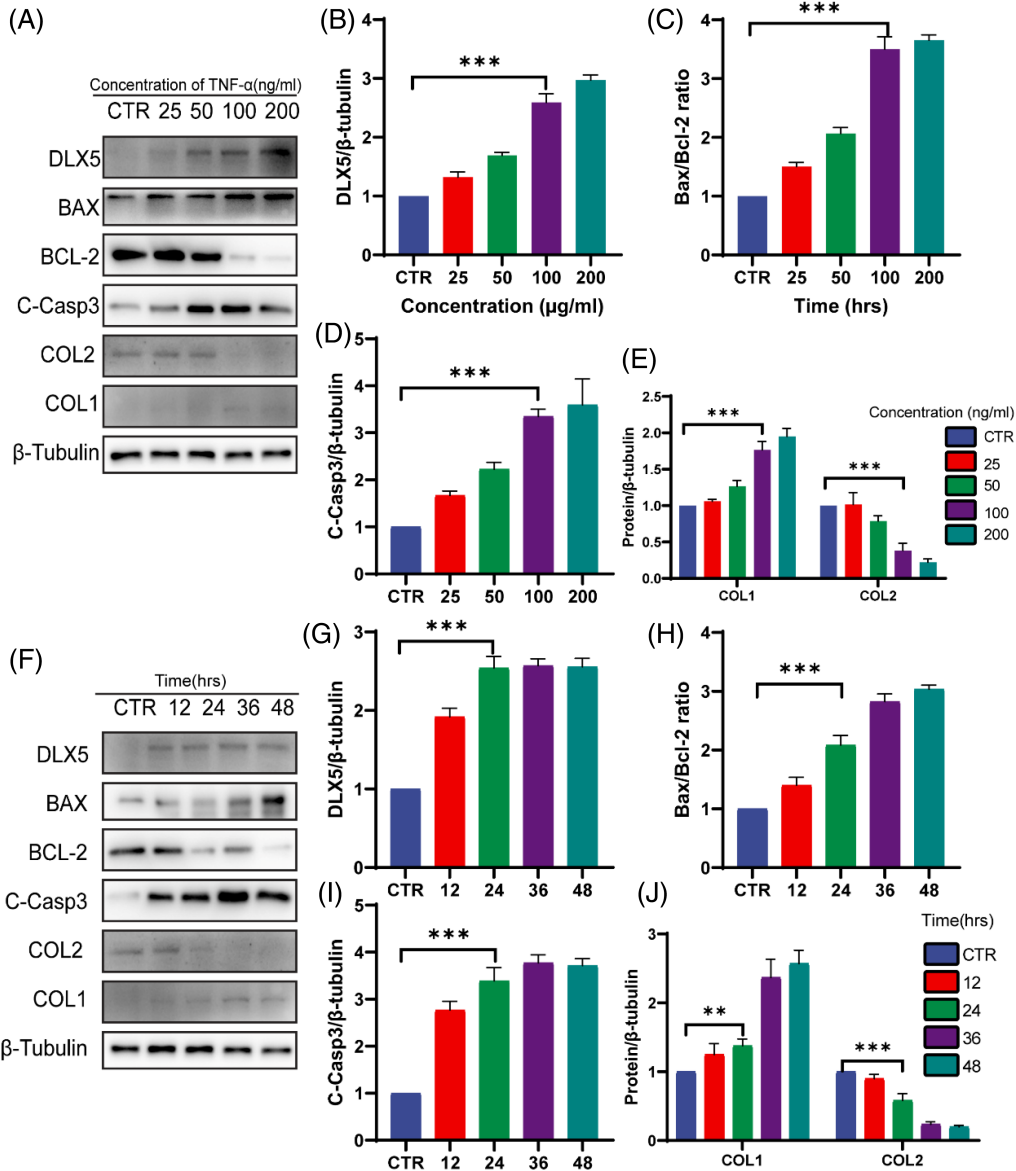
The expression of DLX5 was increased in degenerative NP cells in vitro. (A, F) Representative images of western blots showing changes in DLX5, apoptosis‐related proteins (cleaved Caspase3, Bax/Bcl2), Collagen II and Aggrecan in a dose‐dependent manner (0, 25, 50, 100, 200 ng/mL, 24 h) and time‐dependent (100 ng/mL, 0, 12, 24, 36, 48 h) effects in NP cells. (B–E, G–J) ImageJ software determined the band intensities normalized to those of β‐tubulin. C‐Casp3: cleaved caspase 3. The data are expressed as the means ± SDs (*n* = 3); **p < 0.01; ***p < 0.001.

We constructed a model of NP cell degeneration as widely reported in previous studies.^11^, ^19^ Western blot and quantitative analyses revealed that the expression of DLX5 was affected by TNF‐α treatment in a dose‐ and time‐dependent manner (Figure 3A,B,F,G). Moreover, the expression of the proapoptotic protein BAX increased and the expression of the antiapoptotic protein BCL‐2 decreased, with a corresponding increase in the BAX/BCL‐2 ratio. Additionally, the expression of the caspase 3 activation component, cleaved caspase‐3 (C‐Casp3), increased (Figure 3A,C,D,F,H,I), indicating that TNF‐α treatment increased the degree of apoptosis. In addition, the expression of COL1, a degeneration‐related extracellular matrix indicator, was increased, whereas the expression of COL2, a protective indicator, was decreased (Figure 3A,E,F,J). These results suggest that DLX5 is highly expressed in degenerating NP cells and is accompanied by increased expression of apoptotic markers and an imbalance in the metabolic breakdown of the extracellular matrix. Therefore, a 100 ng/mL concentration for 24 h was used in subsequent experiments.

### 2.4 si‐DLX5 suppressed apoptosis in NP cells

**FIGURE 4:**
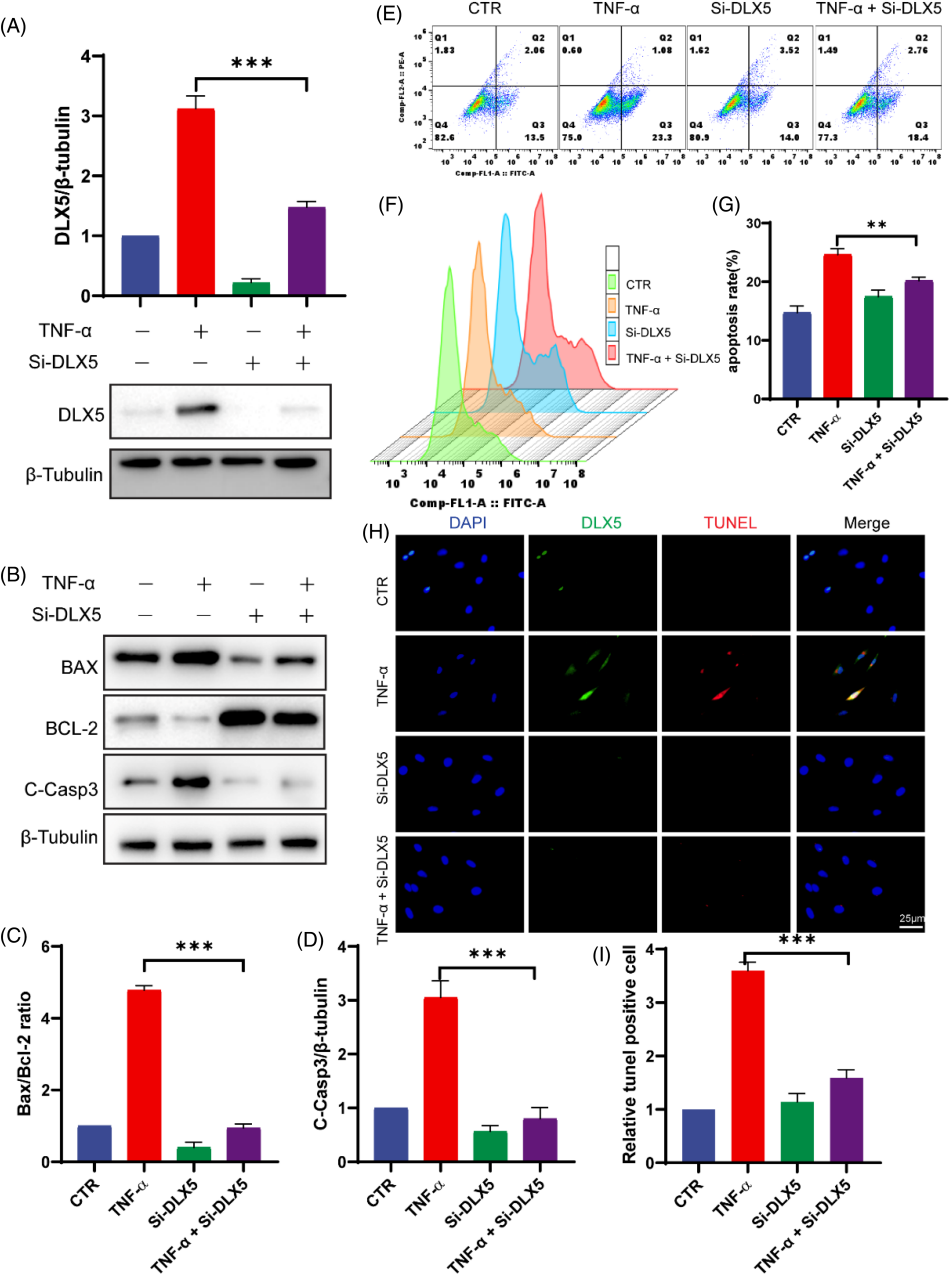
Si‐DLX5 prevented apoptosis in NP cells. (A) Western blot of DLX5 expression and corresponding quantitative analysis normalized to β‐tubulin. (B–D) The protein expression of cleaved caspase‐3, Bax, and Bcl2 in the different treatment groups was determined via western blotting, and quantitative analysis was performed. (E–G) Representative images of flow cytometry data in a dot plot and histogram plot with quantitative analysis of the percentage of annexin‐V‐positive cells. (H, I) TUNEL staining of the different treatment groups is shown, and quantitative analysis was performed (scale bar = 25 μm). The data are expressed as the means ± SDs (*n* = 3); **p < 0.01; ***p < 0.001.

To investigate the role of DLX5 in IVDD, NP cells were transfected with si‐DLX5 to knock down the expression of DLX5 in the NP. First, we used Western blotting to verify that si‐DLX5 successfully knocked down DLX5 48 h post‐transfection (Figure 4A). We subsequently examined the changes in apoptosis‐related indicators after DLX5 knockdown via Western blotting, revealing that the knockdown of DLX5 in TNF‐induced degenerative NP cells significantly decreased the levels of the degenerative indicators BAX/BCL2 ratio and C‐Casp3 (Figure 4B,D). This suggested that si‐DLX5 may play a role in preventing apoptosis in degenerated NP cells. Moreover, we examined the percentage of apoptotic NP cells by flow cytometry, and the results revealed that the percentage of apoptotic cells was significantly reduced after si‐DLX5 treatment (Figure 4E–G). In addition, costaining of NP cells with DLX5 and TUNEL revealed that the knockdown of DLX5 was accompanied by a decrease in TUNEL‐positive cells (Figure 4H,I). These results demonstrated that Si‐DLX5 could effectively reduce the degree of apoptosis in NP cells, suggesting that DLX5 may be involved in IVDD by inducing apoptosis in NP cells.

### 2.5 Melatonin decreases NP cell apoptosis by activating the TGF/Smad2/3 pathway

**FIGURE 5:**
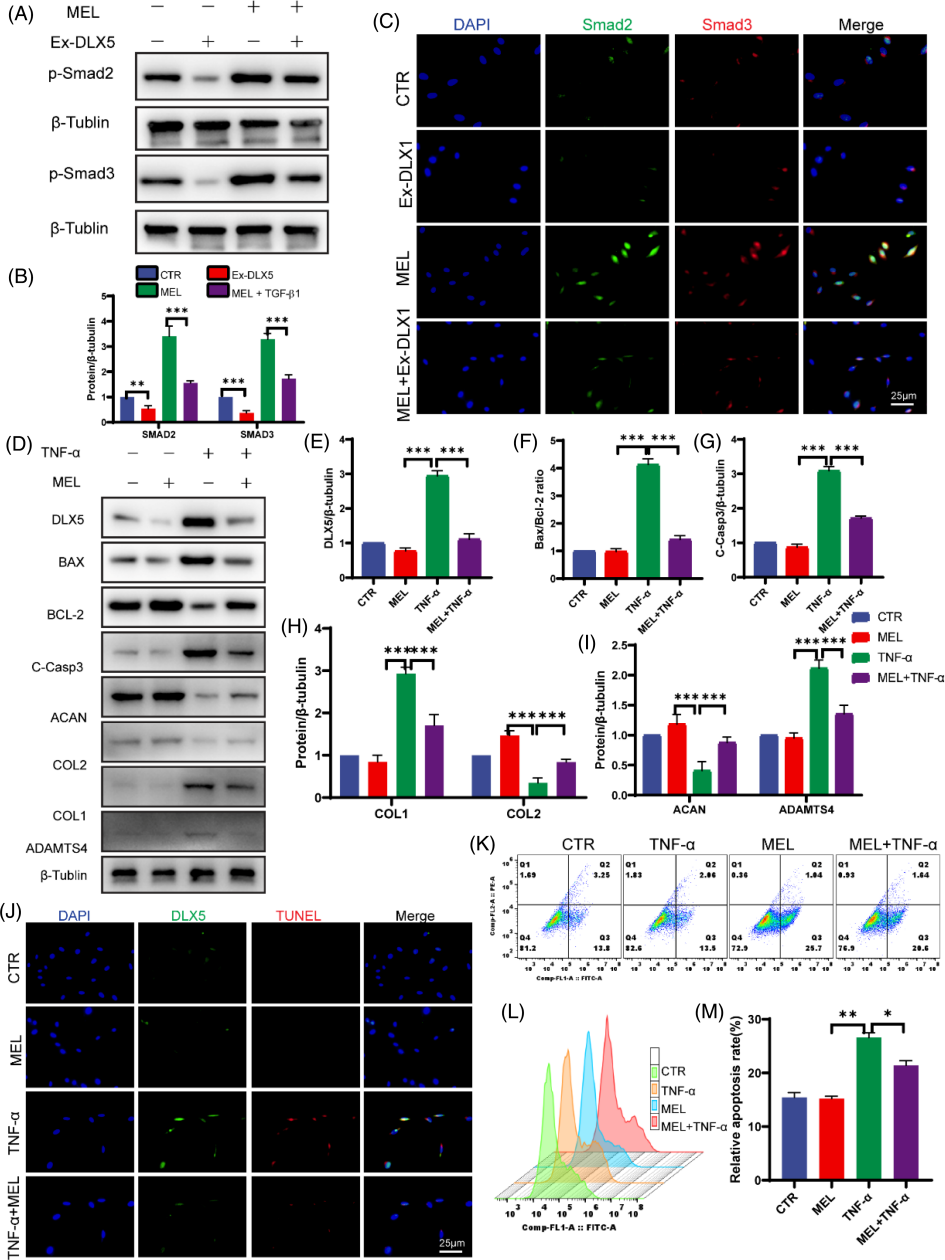
Melatonin decrease NP Cell apoptosis via activating TGF/Smad2/3 Pathway. (A) As determined by western blotting, p‐Smad2, and p‐Smad3 levels after melatonin (2 μM, 12 h) treatment in Ex‐DLX5 or CTR group NP cells. p‐Smad2: phosphorylated Smad‐2; p‐Smad3 phosphorylated: Smad‐3. (B) ImageJ software was used to determine the band intensities, which were normalized to β‐tubulin for Figure 5A. (C) Representative immunofluorescence images are shown to visualize the expression of p‐Smad2 and p‐Smad3 in NP cells (scale bar = 25 μm). (D–I) Protein expression levels and quantitative analysis of DLX5, BAX/BCL2, C‐Casp3, ACAN, COL2, COL1, and ADAMTS4 in groups treated with TNF‐α (100 ng/mL, 24 h) or MEL (2 μM, 12 h) by Western blot. (J) Representative images of DLX5 and TUNEL in different treatment groups (scale bar = 25 μm). (K–M) Representative images of flow cytometry data in a dot plot and histogram plot with quantitative analysis of the percentage of annexin‐V‐positive cells. The data are expressed as the means ± SDs (*n* = 3); *p < 0.05; **p < 0.01; ***p < 0.001.

Previous bioinformatics analyses revealed that DLX5 may regulate the TGF/Smad2/3 pathway. According to previous studies, MEL can activate the TGF/Smad2/3 signaling pathway to regulate cellular functions, including increasing cell proliferation, decreasing apoptosis, and reducing cellular inflammatory responses.^20^, ^21^ For this reason, this study proposes using MEL as a TGF/Smad2/3 activator to conduct follow‐up studies. To further verify the regulatory relationship between DLX5 and TGF/Smad2/3, we constructed a DLX5 overexpression plasmid (Ex‐DLX5) and transfected it into NP cells. MEL (2 μM, 12 h) was used to treat ex‐DLX5 transfected NP cells and empty NP cells. DLX5 overexpression significantly inhibited the phosphorylation level of Smd2/3, a major subunit of the TGF/Smad2/3 pathway. In contrast, the level of Smad2/3 phosphorylation in NP cells after Ex‐DLX5 treatment was significantly increased after MEL treatment (Figure 5A,B), which was verified by cell fluorescence (Figure 5C). These results suggest that DLX5 might affect cell function by inhibiting Smad2/3 phosphorylation, which is reversible by MEL.

To summarize the above results, we hypothesized that MEL may alleviate IVDD by inhibiting DLX5 expression, activating the TGF/Smad2/3 pathway, and reducing apoptosis. To test this hypothesis, NP cells were pretreated with MEL, and the results of Western blot analysis and quantitative analysis revealed that MEL reduced the TNF‐α‐induced increase in DLX5 in a degenerating cell model (Figure 5D,E). This increase was accompanied by a decrease in the apoptotic index BAX/BCL‐2 and a rebound in the expression of ACAN and COL2. However, COL1 and ADAMTS4 expression was decreased in the regression‐related indices (Figure 5D–I). Similarly, in DLX5 and TUNEL costaining of NP cells, the number of DLX5‐positive and TUNEL‐positive cells increased with degeneration, which was rescued by MEL (Figure 5J). The percentage of Annexin‐V‐positive cells determined via flow cytometry showed the same trend (Figure 5K–M). In conclusion, DLX5 may induce apoptosis by inhibiting the TGF/Smad2/3 pathway, leading to IVDD, whereas MEL may have a therapeutic effect.

### 2.6 Melatonin downregulated DLX5 to mitigate NP cell apoptosis in vivo

**FIGURE 6:**
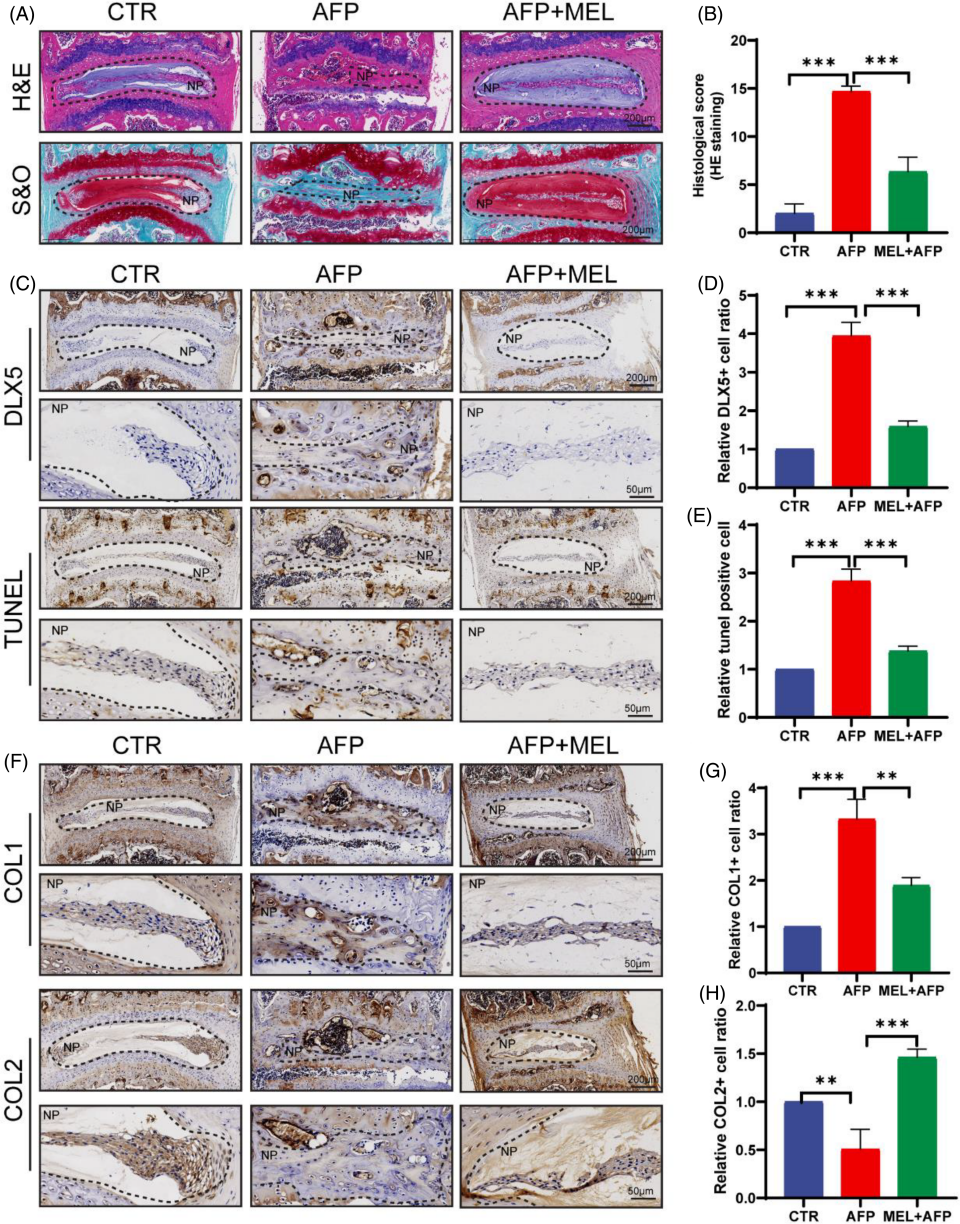
Melatonin downregulated DLX5 to prevent NP cell apoptosis in vivo. (A) Representative H&E and S&O staining images of the CTR, AFP, and AFP + MEL groups. CTR: control (scale bar = 200 μm); AFP: annulus fibrosus puncture group; AFP + MEL: MEL treatment after annulus fibrosus puncture group. (B) Histological score of H&E‐stained samples. (C, D) IHC staining and quantitative analysis of DLX5+ and TUNEL+ NP tissues from different groups (scale bar = 200 μm upper panels and 50 μm lower panels). (F–H) IHC staining of COL1 and COL2 in mouse NP tissues and IVDs; corresponding quantitative analysis was performed (scale bar = 200 μm upper panels and 50 μm lower panels). The data are expressed as the means ± SDs (*n* = 3); *p < 0.05; **p < 0.01; ***p < 0.001.

To verify the potential molecular mechanism by which MEL reduces DLX5 to slow IDD progression, MEL was intraperitoneally injected into AF puncture‐induced IVDD model mice. The mice were randomly divided into 3 groups: the control group (CTR), the AF puncture group (AFP), and the AFP + MEL treatment group. The mice in each group were sacrificed after 8 weeks of treatment. H&E and S&O staining revealed that the disc structure was disordered in the AF puncture group, and the boundary of the NP tissue was not clear; however, it was similar in the CTR and AF + MEL groups, and the disc structure tended to be typical in the AF + MEL group, although there was a reduction in the number of nucleus pulposus cells (Figure 6A,B). Moreover, immunohistochemical staining of the intervertebral discs of the three groups of mice revealed a significant decrease in the expression level of DLX5 relative to the AF group, which was close to the normal level (Figure 6C,D). The TUNEL staining results were similar, suggesting a decrease in the percentage of apoptotic NP cells (Figure 6C,E). Notably, the results of disc degeneration marker staining revealed that MEL significantly decreased the expression of COL1 and restored the expression of COL2 (Figure 6F,G). In conclusion, MEL might reduce the degree of NP cell apoptosis and alleviate disc degeneration by inhibiting DLX5.

## 3 METHODS

### 3.1 Bioinformatics analysis

The public database used in this study was Affymetrix® whole genome gene chip microarrays GSE70362, which contains NP tissue samples data. The data type is whole‐genome gene chip results. Differential genes with *p* < 0.05 and Foldchange >2 were selected for further analysis. The volcano plot and Circos plot were analyzed using the ggPlot2 package, while the Sankey plot was drawn using the ggplot2 package and ggalluvial package. The protein–protein interaction network was constructed using the STITCH website. For database information, see Table S1.

### 3.2 Patient samples collection

As part of this study, tissues were collected from The Third Affiliated Hospital of Guangzhou University of Chinese Medicine, approved by its ethics committee, and informed consent was obtained. Nucleus pulposus tissues were obtained from patients with intervertebral disc degeneration during the surgery. According to magnetic resonance imaging findings, intervertebral disc degeneration was indicated by a decrease in signal intensity and a narrowing of disc space according to the five‐grade Pfirrmann system. For sample information, see Table S2.

### 3.3 Culture of NP cells

The obtained NP tissue was washed three times with cold PBS buffer, chopped, and digested three times following collagenase II (0.5 mg/mL) and pancreatin (0.3 mg/mL) for 20 min. A fresh media DMEM was then added to the cells, and they were further incubated for 72 h at 37°C, 5% CO_2_. Cells were passaged at 80% confluency. The subsequent experiments used cells in the logarithmic growth phase at passages 3–5.

### 3.4 IVDD mice model

A murine IVDD model was established via surgery performed under aseptic conditions as described studies.^26^ Male C57BL/6 mice weighing approximately 25 g and that were 8–9 weeks old (*n* = 3 per group) were raised in SPF environment under constant temperature (23–25°C) and humidity (50%) with a 12‐h light/12‐h dark circadian cycle. The mice were anesthetized with 3% pentobarbital sodium and then supine fixed on the surgical table. The abdominal fur was shaved, and the skin was disinfected. The psoas major muscle was exposed by pining intestinal loops under a microscope. Then, expose IVDs by moving head‐ward along the psoas major muscle. IVDD model was established in mice by puncturing their discs with a 27G needle and 1.0 mm insertion at lumbar vertebrae 3–4 and 4–5; defective annulus fibrosus was established after the disc.

Melatonin (MEL) was diluted to 5 mg/mL with normal saline. The mice in the MEL plus AF puncture group were intraperitoneally injected with MEL at various concentrations (0, 1, 3, or 5 mg/kg/week), and 2 mg/mL resulted in the greatest improvement in IVDD. One week after the operation, 2 mg/kg/week of MEL was intraperitoneally injected into the mice for three consecutive weeks. Mice in the control group received no treatment following surgery.

### 3.5 RNA isolation, cDNA synthesis, and RT‐qPCR

RT‐qPCR analysis was performed after extracting RNA and synthesizing first‐strand cDNA using SYBR Green Master Mix (Takara Bio, Otsu, Japan) in accordance with the manufacturers instructions. For primers information, see Table S3.

### 3.6 Immunohistochemical staining

Sections of tissue were baked in a 60°C incubator for 1 h, then overlaid with Xylene to dewax, hydrated with gradient alcohol, microwaved to repair antigens, and treated with 3% hydrogen peroxide. After pre‐blocking, the slices were incubated with primary antibodies overnight at 4°C, followed by 30 min of secondary antibody incubation with either anti‐rabbit‐HRP or anti‐mouse‐HRP. Signal detection was achieved using DAB, and tissue was counterstained with hematoxylin.

### 3.7 HE and Safranin‐O staining

The tissue sections were initially dewaxed and hydrated, followed by staining with hematoxylin solution to visualize the nuclei, which appeared blue. After rinsing in water and differentiation in acid alcohol, counterstaining was performed using eosin solution to highlight the cytoplasm and extracellular matrix components, which appeared pink. For Safranin‐O staining, the sections were dewaxed and hydrated, then stained with safranin‐O solution to specifically stain cartilage and connective tissue red. Subsequently, differentiation was carried out in acid alcohol. Finally, the sections were dehydrated, cleared, and mounted onto slides for further analysis. The histological scoring criteria were adopted from previous studies in the field.^27^

### 3.8 Immunofluorescence analyses

After being immersed in 75% alcohol for 5 min, the cell climbing slices were wrapped in sterilized membranes and placed in a 24 well plate. Cells were transferred to 24‐well plates at 30% confluency. Fixed NP cells were permeabilized with 0.1% Triton X‐100, blocked with 1% BSA, and incubated with primary and secondary antibodies. Fluorescence microscopy was used to observe the anti‐fluorescence quenching sealing tablets.

### 3.9 SiRNA transfection

According to the manufacturer's protocol, siRNA reagents were transfected for 48 h into cells using X‐tremeGENE siRNA Transfection Reagent (Roche, Switzerland). For SiRNA information, see Table S4.

### 3.10 Flow cytometry

Annexin V/PI Double Staining The cells were trypsinized and washed three times with PBS, centrifuged (2000 rpm at room temperature) for 2 min, adjusted to 5 × 10^4^/ml and double‐stained with annexin V‐FITC and PI (Annexin V‐FITC Apoptosis Detection Kit, BD Biosciences). For regents information, see Table S5.

### 3.11 Western blotting

Forty gram of total proteins were electrophoresed on SDS‐PAGE and transferred to PVDF membranes (Millipore, Massachusetts). After pre‐blocking, the blots were incubated with primary antibodies overnight at 4°C, followed by 30 min of secondary antibody incubation with either anti‐rabbit‐HRP or anti‐mouse‐HRP. Enhanced chemiluminescence was used to detect the signals.

### 3.12 Statistical analysis

Statistical analyses were performed using SPSS 23.0 software (IBM). One‐way analysis of variance and Student's *t* test were used to analyze the differences between groups. Typically, the results are presented as the mean ± SD, and *p* < 0.05 was considered statistically significant.

## 4 DISCUSSION

Low back pain (LBP) is a common aging‐associated disease and a leading cause of disability. The main driver of LBP is IVDD.^2^ Current treatments for IVDD, such as analgesics and anti‐inflammatory drugs, focus mainly on pain management without slowing the progression of the disease.^28^ New therapeutic strategies that can slow or even reverse disease progression are urgently needed.

There is increasing evidence that DLX5 induces inflammation and is implicated in many pathogenic mechanisms and diseases, such as cancer, neurodevelopmental disorders, pregnancy syndrome, osteogenesis, and arthritis.^15^, ^28^, ^29^, ^30^, ^31^, ^32^ Inflammation combined with imbalanced ECM synthesis are the main features associated with the development and progression of IVDD.^10^, ^11^ In this study, we first identified DLX5 as a key gene involved in IVDD at the cellular, tissue, and organismal levels. DLX5 is involved in TNF‐α‐induced proapoptotic responses in addition to anabolism and catabolism imbalances in the ECM. In addition, we also found that si‐DLX5 can inhibit apoptosis and ECM degradation. Therefore, DLX5 may aggravate IVDD by inducing apoptosis and ECM degradation. The TGF/Smad2/3‐associated signaling pathway was subsequently identified as a target of DLX5 via GO analysis. Next, as reported in previous studies, we used MEL to activate the TGF/Smad2/3 pathway.^20^, ^21^ We investigated the effect of DLX5 on mediating the TGF/Smad2/3 signaling pathway in NP cells. These results indicated that DLX5 could inhibit the phosphorylation of Smad2/3, whereas MEL promoted the phosphorylation of Smad2/3. Furthermore, MEL partially reversed Ex‐DLX5‐mediated suppression of p‐Smad2/3 expression in NP cells, thereby reducing TNF‐α‐induced NP cell apoptosis and ECM catabolism. Similarly, DLX5 disrupted the MEL‐mediated promotion of Smad2/3 phosphorylation. These results indicate that MEL inhibits the progression of IVDD by activating the TGF/Smad2/3‐associated signaling pathway, reversing the effects of DLX5 in inducing apoptosis, inhibiting ECM anabolism, and enhancing ECM catabolism. Furthermore, these conclusions have been validated by in vivo experiments.

Dlx5 is a nuclear transcription factor that plays important roles in embryogenesis, organ development, tissue differentiation, and bone formation.^29^, ^30^, ^31^, ^32^ In addition, DLX5 affects cellular functions such as inflammation, proliferation, and apoptosis.^24^, ^26^, ^33^ In this study, DLX5 was identified via microarray analysis as one of the most upregulated differentially expressed genes in NP tissues from IVDD patients. Numerous studies have confirmed that DLX5 plays a key role in OA.^15^, ^29^ However, the unique role of DLX5 in regulating IVDD progression remains unclear. Previous studies have shown that DLX5 is upregulated in BMP‐treated NP cells. In this study, we found that DLX5 is upregulated in degenerated NP cells, samples, and animal models of IVDD. Furthermore, RT–qPCR, Western blotting, and IHC were used to analyze patient samples, and we demonstrated that the expression level of DLX5 was positively correlated with the degree of disc degeneration. DLX5 is significantly increased in a degenerative NP cell model. The level of apoptosis in degenerative NP cell models transiently transfected with si‐DLX5 was significantly lower than in controls. Collectively, these findings suggest that DLX5 is a potential therapeutic target for IVDD.

Melatonin (N‐acetyl‐5‐methoxytryptamine) is synthesized by the pineal gland and many other organs.^34^ MEL was identified because of its roles in regulating tissue homeostasis, circadian rhythms, embryonic development, and regeneration.^35^, ^36^ Previous studies have identified MEL as a Smad2/3 activator.^37^, ^38^ The TGF/Smad2/3 signaling pathway plays a key and unique role in the occurrence and development of IVDD.^43^ Chen et al. studied TGF/Smad2/3 signaling in intervertebral disc health and disease and reported that TGF/Smad2/3 pathway signaling inhibits ECM degradation, increases ECM synthesis, promotes cell proliferation, inhibits cell death, and reduces inflammatory responses.^44^ Activation of TGF/Smad2/3 signaling in cartilage maintains the cellular phenotype and tissue homeostasis through the Smad2/3 pathway, resulting in ligand‐induced transcription that increases the expression of aggrecan, Col2, TIMP‐3, and Sox9.^44^ Previous studies have shown that TGF/Smad2/3 alleviates IVDD by inhibiting syndecan‐4 expression.^45^ Having elucidated the role of DLX5 in IVDD, we propose that DLX5 is involved in the phosphorylation of Smad2 and Smad3. In the present study, we showed that DLX5 partially regulates IVDD by promoting apoptosis and altering the ECM composition by inhibiting the TGF/Smad2/3 signaling pathway. In the present study, we explored the relationship between DLX5 and the inflammatory response of NP cells and found that the expression of DLX5 was upregulated in TNF‐α‐treated NP cells. Therefore, we hypothesized that DLX5 might regulate IVDD by suppressing the inflammatory response. Further studies are needed to confirm this hypothesis.

Although the therapeutic effect of MEL in a surgically induced IVDD mouse model is promising, the optimal dose remains to be determined, and the side effects remain unknown. Several questions still need to be addressed, and many obstacles must be overcome before this treatment can be applied to humans. Furthermore, the exact mechanism by which DLX5 levels increase during degeneration remains unclear. However, further studies are needed to elucidate the role of DLX5 in IVDD fully. Additionally, more attention should be given to how this dataset fits into previous studies investigating the role of TNF‐alpha in the progression of disc degeneration and the relative advantages/disadvantages of targeting DXL5 over other strategies, such as direct TNF‐alpha targeting or receptor targeting.

Taken together, our findings demonstrate that DLX5 expression positively correlates with the severity of IVDD and that treatment with MEL significantly attenuates IVDD in a surgically induced mouse model of IVDD. For the first time, our study links DLX5 to the TGF/Smad2/3 signaling pathway and demonstrates the molecular mechanisms regulating IVD maintenance and destruction. In summary, our results provide a potential therapeutic target for alleviating IVDD‐related NP cell apoptosis and inflammation‐induced changes in the ECM composition.

## AUTHOR CONTRIBUTIONS

**Kuibo Zhang**: Project administration; investigation; writing – original draft; visualization. **Hua Wang**: Methodology; data curation; formal analysis. **Ling Mo**: Validation; resources. **Xiaohui Huang**: Software. **Chao Yuan**: Writing – review & editing. **Caijun Liu**: Conceptualization; supervision.

## CONFLICT OF INTEREST STATEMENT

The authors declare no conflicts of interest.

## Supporting information


**Table S1.** Information of microarray dataset.
**Table S2**. Information of human disc samples from 25 patients.
**Table S3**. Primer sequences.
**Table S4**. Information of si‐RNA target sequence.
**Table S5**. Information of main regents.

## Data Availability

All data are available in the main text or the materials.
