# Supplementary material for: Melatonin attenuates degenerative disc degression by downregulating DLX5 via the TGF/Smad2/3 pathway in nucleus pulposus cells
Source: JOR Spine. 2024 Nov 13;7(4):e70014. doi: 10.1002/jsp2.70014 (PMC11558270; doi:10.1002/jsp2.70014)
Supplement: Supplementary file 1 — Table S1. Information of microarray dataset. Table S2. Information of human disc samples from 25 patients. Table S3. Primer sequences. Table S4. Information of si‐RNA target sequence. Table S5. Information of main regents. [file JSP2-7-e70014-s001.docx]

Supplementary Tables

**Table S1. Information of microarray dataset**

| **Dataset** | **Platform** | **Samples** | **RNA** | **Year** | **Organism** | **tissue** |
| --- | --- | --- | --- | --- | --- | --- |
| GSE70362 | GPL17810 | 48 | mRNA | 2015 | Homo sapiens | intervertebral disc |

**Table S2. Information of human disc samples from 25 patients for molecular biology experiments**

| Pfirrmann grade |  | | Sex | |  | |  | | Intervertebral disc level | | | | | Total |
| --- | --- | --- | --- | --- | --- | --- | --- | --- | --- | --- | --- | --- | --- | --- |
|  |  | Male | | Female |  | L1/2 | | L2/3 | | L3/4 | L4/5 | L5/S1 | thoracic intervertebral disc |  |
| I |  | 2 | | 3 |  | 0 | | 0 | | 1 | 1 | 0 | 3 | 5 |
| II |  | 2 | | 3 |  | 0 | | 1 | | 1 | 2 | 0 | 1 | 5 |
| III |  | 2 | | 3 |  | 0 | | 0 | | 1 | 1 | 3 | 0 | 5 |
| IV |  | 3 | | 2 |  | 0 | | 0 | | 0 | 2 | 3 | 0 | 5 |
| V |  | 4 | | 1 |  | 0 | | 0 | | 1 | 0 | 4 | 0 | 5 |
| Total |  | 13 | | 12 |  | 0 | | 1 | | 4 | 6 | 10 | 4 | 25 |

**Table S3:** **Primer sequences**

| Gene | Forword sequence (5’-3’) | Reverse sequence (5’-3’) | Accession no. |
| --- | --- | --- | --- |
| DLX5  (Human) | TTCCAAGCTCCGTTCCAGAC | GAATCGGTAGCTGAAGACTCG | [NM_005221](http://www.ncbi.nlm.nih.gov/entrez/query.fcgi?cmd=Search&db=Nucleotide&term=NM_005221) |
| β-tubulin  (Human) | TGGACTCTGTTCGCTCAGGT | TGCCTCCTTCCGTACCACAT | [NM_178014](http://www.ncbi.nlm.nih.gov/entrez/query.fcgi?cmd=Search&db=Nucleotide&term=NM_178014) |

**Table S4: Information of si-RNA target sequence**

| Species | Si-RNA | Target sequence (5’-3’) |
| --- | --- | --- |
| Human | Si-DLX5_01 | GGAGACAGAGACUUCACGACU |
| Human | Si-DLX5_02 | CGAUGACAGGAGUGUUUGACA |
| Human | Si-DLX5_03 | GAGUCUUCAGCUACCGAUUCU |

**Table S5: Information of antibodies**

| Antibody | Source | Cat# No. | WB | IHC | IF |
| --- | --- | --- | --- | --- | --- |
| Primary antibody |  |  |  |  |  |
| DLX5 | Abcam | ab109737 | 1:1000 | 1:100 | 1:100 |
| BAX | Abcam | Ab32503 | 1:1000 |  |  |
| BCL2 | Abcam | Ab182858 | 1:1000 |  |  |
| Cleaved Caspase 3 | Abcam | ab32043 | 1:1000 |  |  |
| COL 2 | Bioss Antibodies | Bs-10589R | 1:1000 | 1:100 | 1:50 |
| COL 1 | Bioss Antibodies | bs-10423R | 1:1000 | 1:100 | 1:100 |
| Beta Tubulin (HRP conjugated) | Bioss Antibodies | bsm-52847R | 1:5000 |  |  |
| p-Smad2 | Abcam | ab280888 | 1: 1000 | 1:200 |  |
| p-Smad3 | Abcam | ab52903 | 1: 1000 | 1:200 |  |
| ACAN | Cell Signaling Technology | 3033 | 1:1000 | 1:200 | 1:100 |
| ADAMTS4 |  |  |  |  |  |
|  |  |  |  |  |  |
| Secondary antibody |  |  |  |  |  |
| Anti-rabbit IgG, HRP-linked Antibody | Cell Signaling Technology | 7074S | 1:5000 |  |  |
| Anti-mouse IgG, HRP-linked Antibody | Cell Signaling Technology | 7076S | 1:5000 |  |  |
| Goat anti-Rabbit IgG (H+L) Cross-Adsorbed Secondary Antibody, Alexa Fluor™ 488 | Thermo Fisher Scientific | A-11008 |  |  | 1:2000 |
| Goat anti-mouse IgG (H+L) Secondary Antibody, DyLight™ 488 | Thermo Fisher Scientific | A-10680 |  |  | 1:2000 |
| Goat anti-Rabbit IgG (H+L) Highly Cross-Adsorbed Secondary Antibody, Alexa Fluor™ 555 | Thermo Fisher Scientific | A-21429 |  |  | 1:2000 |
| Goat anti-Rabbit IgG (H+L) Cross-Adsorbed Secondary Antibody, Alexa Fluor™ 594 | Thermo Fisher Scientific | R-37117 |  |  | 1:2000 |
| Goat anti-Rabbit IgG (H+L) Highly Cross-Adsorbed Secondary Antibody, Alexa Fluor™ 647 | Thermo Fisher Scientific | A-21245 |  |  | 1:2000 |
| Reagents |  |  | **Application** |  |  |
| PrimeScript™ II Reverse Transcriptase | TaKaRa | 2690A | RT-qPCR |  |  |
| TB Green® Premix Ex Taq™ II (Tli RNaseH Plus) | TaKaRa | RR820A | RT-qPCR |  |  |
| DAKO REALTM EnVision^TM^ Detection system | DAKO | K5007 | IHC |  |  |
| Lipofectamine™ 3000 Transfection Reagent | Invitrogen, Thermo Fisher Scientific | L3000015 | Si-RNA, plasmid transfection |  |  |
